# Therapeutic Targets for Regulating Oxidative Damage Induced by Ischemia-Reperfusion Injury: A Study from a Pharmacological Perspective

**DOI:** 10.1155/2022/8624318

**Published:** 2022-04-11

**Authors:** Walter Ángel Trujillo-Rangel, Leonel García-Valdés, Miriam Méndez-del Villar, Rolando Castañeda-Arellano, Sylvia Elena Totsuka-Sutto, Leonel García-Benavides

**Affiliations:** ^1^Departamento de Ciencias Biomédicas, Centro Universitario de Tonalá, Universidad de Guadalajara, C.P. 45425, Tonalá, Jalisco, Mexico; ^2^Departamento de Fisiología, Centro Universitario de Ciencias de la Salud, C.P. 44340, Guadalajara, Jalisco, Mexico

## Abstract

Ischemia-reperfusion (I-R) injury is damage caused by restoring blood flow into ischemic tissues or organs. This complex and characteristic lesion accelerates cell death induced by signaling pathways such as apoptosis, necrosis, and even ferroptosis. In addition to the direct association between I-R and the release of reactive oxygen species and reactive nitrogen species, it is involved in developing mitochondrial oxidative damage. Thus, its mechanism plays a critical role via reactive species scavenging, calcium overload modulation, electron transport chain blocking, mitochondrial permeability transition pore activation, or noncoding RNA transcription. Other receptors and molecules reduce tissue and organ damage caused by this pathology and other related diseases. These molecular targets have been gradually discovered and have essential roles in I-R resolution. Therefore, the current study is aimed at highlighting the importance of these discoveries. In this review, we inquire about the oxidative damage receptors that are relevant to reducing the damage induced by oxidative stress associated with I-R. Several complications on surgical techniques and pathology interventions do not mitigate the damage caused by I-R. Nevertheless, these therapies developed using alternative targets could work as coadjuvants in tissue transplants or I-R-related pathologies

## 1. Introduction

Ischemia-reperfusion (I-R) injury is a cellular phenomenon caused by the interruption of oxygen flow and the consecutive restoration of oxygen concentration, which is known as reperfusion [1]. The reperfusion of ischemic tissues subjected to arterial occlusion causes the formation of a characteristic lesion that accelerates apoptosis and necrosis development [2]. I-R occurs in individuals with multiple pathologies and those receiving an intervention. Thus, it is inevitable in different conditions, such as cardiac, thoracic, and peripheral vascular diseases, and interventions, including major vascular surgery and solid organ transplantation [3–5]. Although the prevalence of ischemia is high, the treatment and preventive strategies for this lesion are not standardized or, simply, not effective enough to resolve damage [6]. Due to the impact of this condition on health systems and its epidemiological distribution, preventive pharmacological strategy is needed urgently. Even when effective therapy is necessary, I-R injury is still poorly understood, and researchers are looking for alternatives or relevant molecular targets that can modulate damage induced by this injury [7]. To date, I-R is characterized by an augmented inflammatory reaction that increases the expression of reactive oxygen species and reactive nitrogen species, which exacerbate tissue damage [8]. Hypoxia-inducible factors (HIF) are oxygen-regulated transcription factors that play important roles in the detection and adaptation of hypoxia [9]. Besides, they act as critical effectors in response to reduced oxygen levels and have a large number of genes under their control [10]. The expression of HIF-1*α* together with the generation of mitochondrial reactive oxygen species (ROS) is reinforced in response to ischemic oxidative stress [11]. In hypoxia, HIF-1*α* stabilizes by the accumulation of elevated levels of ROS generated from complex III in the mitochondria [11]. The mechanism behind this is oxidative inactivation of nonheme iron at the catalytic site of the enzyme prolyl hydroxylase [12]. Notably, ROS driven by hypoxia activates NF-kB and other transcription factors such as nuclear factor erythroid 2-related factor 2 (NrF2), which plays a vital role in the regulation of protein transcription involved in antioxidant defense [13]. The mitogen-activated protein kinase (MAPK) pathway has important implications as it interacts with ROS, which leads to a higher expression of vascular endothelial growth factor (VEFG) [14, 15]. The increased expressions of vascular endothelial growth factor (VEGF) and its receptors VEGF-R1 and R2 play a part in the activation of HIF-1*α* by ROS, and they have fundamental roles in maximizing cell survival [16]. Moreover, ROS activates other intracellular signaling pathways including MAPK, NF-kB, and upstream of MMP [17]. In addition, mitochondrial ROS can enhance damage via different mechanisms, such as mitochondrial permeability induction, ROS-mediated inflammatory and proapoptotic signaling, extracellular remodeling, and primarily oxidative damage in structures and intramitochondrial molecules, which contribute to the development of I-R lesion [18]. The therapeutic value of mitochondrial ROS attenuation in modulating I-R damage to the cell must be emphasized. Hence, effective therapeutic alternatives for ischemic reconditioning and tissue preparation for a possible ischemic event can be developed [19]. Research continues its course. However, certain points must be clarified, and the active principles and crucial receptors that can be an alternative for modulating this phenomenon should be determined [20].

The I-R phenomenon is poorly understood and highly variable between tissues. Although multiple mechanisms are known day by day, there is no effective therapy in clinical phases to date [21]. However, the design of effective therapy is necessary due to the significant relationship between this phenomenon and multiple cardio-obstructive pathologies and surgical procedures [22]. Two main approaches come to light: inflammation and oxidative stress induced by I-R damage [23]. Oxidative stress is very relevant due to the multiple opportunities for damage control. Unfortunately, the mechanisms are not applied. That is the reason for doing this work. Specify and conceptualize the main therapeutic targets towards which the pharmacological designs that allow a resolution of ischemic pathologies should be oriented. Therefore, the current study is aimed at providing ideas and research objectives for resolving oxidative and mitochondrial damage to modulate I-R injury in different tissues.

## 2. Ischemia-Reperfusion

Over the years, the concept of I-R has been changing and developing, thereby making us closer to discovering or establishing effective therapeutic interventions [7]. In I-R injury, triggering mechanisms begin at the time of arterial blood flow interruption in a tissue or organ, which produces an imbalance of metabolic substrates, leading to hypoxia [18]. I-R is a critical clinical condition, and physicians find it challenging to manage as it requires the preservation of tissue or organ function among individuals with different pathologies or those undergoing surgical procedures [6]. However, in clinical practice, the outcomes after reperfusion in ischemic tissues are far from optimal, and numerous damages are induced to the tissues [24]. As a consequence, reoxygenation is correlated with the exacerbation of local tissue injury and severe local or systemic inflammatory response. This was observed in tissues subjected to I-R, which are comparable with the degree of necrosis observed 24 h after permanent ischemia [25]. Cell dysfunction, damage, and death are associated with the magnitude and duration of ischemia. Therefore, blood flow restoration is still based on injury resolution. However, not all tissues or organs respond similarly to ischemic insult; thus, reperfusion is important to improve cell necrosis [26, 27].

## 3. Mitochondrial Oxidative Damage in Ischemia-Reperfusion Injury

Mitochondrial oxidative damage is important for the development of I-R, which is directly correlated with mitochondrial ROS and reactive nitrogen species (RNS) formation [28]. In myocardial infarction, the heart requires substantial amounts of energy from phosphates to maintain function and transport [29]. Nevertheless, ATP must be continually synthesized by the oxidative substrate in the mitochondria, thereby increasing the demand for reactive species formation [22]. The inhibition of electron flow along the respiratory chain leads to energy conservation. In addition, limited oxygen supply can inhibit mitochondrial complex IV, which blocks electron transfer to molecular oxygen and reduces ATP concentrations [30]. During the ischemic phase, ATP concentrations are unsuccessfully maintained by glycolysis; hence, the condition further exacerbates, which leads to lactic acid accumulation. Next, intracellular pH decreases. Simultaneously, the Na^+^/H^+^ antiporter is activated in response to decreased cytosolic hydrogen potential. The cell is overloaded with Na^+^, which cannot be pumped out of the cell by Na/K-ATPase. If the ATP concentrations are low due to decreased inner mitochondrial membrane gradient, FOF1-ATPase hydrolyzes ATP to regulate the condition [31]. Due to the inability of the mitochondria to produce significant amounts of ATP, compensatory anaerobic glycolysis occurs as a resolution mechanism. However, paradoxically, a considerable amount of this ATP will be hydrolyzed by FOF1-ATPase [32]. Concurrently, Na+ can prevent the release of Ca^2+^ by the Na^+ /^Ca^2+^ antiporter, thereby attempting to reverse the process. Calcium could enter the cytosol or even the mitochondria via the reversal of the Na^+/^Ca^2+^ antiporter mechanism [18]. However, the mitochondrial matrix absorbs Ca^2+^ after the reperfusion process via the uniporter, which then overloads the matrix with this ion. The opening of the mitochondrial permeability transition pore (mPTP) is one of the essential mitochondrial mechanisms in I-R. These pores are strictly linked with mitochondrial ROS and RNS release. Mitochondrial permeability allows ions and solutes with a low weight to freely move between the mitochondrial matrixes [33]. The main concept of mPTP was considered as an in vitro artifact without any pathophysiological significance. A previous study has later supported this notion and confirmed their role in the development of some diseases [34]. ROS are some of the main triggers of mPTP opening by overloading the matrix with high Ca^2+^ concentrations. However, there are other factors and molecules implicated in reperfusion. One of them is the influx of oxygen in anoxic cells, which leads to the formation of free radicals, a consequence of respiratory chain inhibition [34]. Almost all free radicals may be produced via the activation of xanthine oxidase. This enzyme is activated in hypoxia during ischemia [35].

In addition to cellular phosphate and depleted adenine nucleotide levels, which are commonly correlated with the ischemia process, high Ca^2+^ concentrations and oxidative stress conditions can activate mPTP [35]. During the reperfusion phase, the pH returns to preischemic insult values. This phenomenon is attributed to the activity of Na^+^/H^+^ antiporter that grants the release of lactic acid, which makes mPTP relevant and facilitates its full ability to exhibit its effect [36].

## 4. Oxidative Molecular Mechanisms Involved in Ischemia-Reperfusion Injury

There are complementary processes that are directly or indirectly correlated with mitochondrial oxidative stress and that play an essential role in the development of I-R injury [37]. Events, such as increased cations at the cytosolic level, mitochondrial injury, formation of oxidative and nitrosative species, transcriptional reprogramming, apoptosis activation processes, autophagy, necrosis, inflammation, immunity-mediated injury, endothelial injury, activation of ferroptosis, and the nonreflux phenomenon, are triggered or enhanced by blood flow obstruction and restoration [38].

### 4.1. Calcium Overload

Calcium overload and cytosolic cation increment are the initial mechanisms activated after the start of ischemia. All tissues and cells affected by this condition become dependent on anaerobic glycolysis ATP supply [39]. However, as an alternative for restoring pH to normal levels, some anticarriers including Na^+^/H^+^ are activated to address the accumulation of cytosolic Ca^2+^. Nevertheless, the expression of cytosolic Ca^2+^ is even higher during reperfusion, when the removal of H^+^ ions of extracellular origin paradoxically raises the proton gradient, thereby accelerating the proton exchange function [40]. All these events and alterations as well as high Ca^2+^ concentrations activate different pathways involved in I-R-induced cell death. Pumping up Ca^+^ directly to the mitochondria via Ca^2+^ uniporters is a mechanism that can help cells manage Ca^+^ overload [41].

### 4.2. Formation of Reactive Oxygen and Nitrogen Species in Mitochondria

ROSs are normally produced in the mitochondria, endoplasmic reticulum, plasma membrane, and cytoplasm during physiological metabolic processes [42]. ROS and RNS production in the cell starts with the reduction of oxygen and nitrogen levels, which are extremely basic and simple reactions. However, they are extremely important in cell function [43]. Generally, the mitochondria are the main source of cellular oxidative and nitrosative stress. Nonetheless, study results that reinforce this argument, particularly in nitrosative stress and heart disease, must be further validated [44, 45]. The mitochondria are involved in reactive species formation, with production directly involved with cytosol reactive species concentrations [46]. Moreover, complexes I and III of the electron transport chain are involved in ROS formation along with NAD^+^-linked oxidoreductases in the mitochondrial matrix. This notion has been reviewed and presented in several studies [42, 47, 48]. The reactive species correlated with the mitochondria and oxidative and nitrosative damage are superoxide molecules, hydrogen peroxide, hydroxyl radicals (-OH), nitric oxide (NO), nitroxyl anion, nitrosonium cation (NO^+^), and peroxynitrite (ONOO-) [49, 50].

### 4.3. Ferroptosis in I-R Phenomenon

Ferroptosis is a type of cell death that is an alternative to apoptosis. It is characterized by the accumulation of iron-dependent lipid hydroperoxides at alarming levels. Moreover, it cannot be inhibited by factors associated with other known types of cell death [51]. Hence, it is morphologically, biochemically, and genetically different from other types of cell death, and it is involved in various pathological events in which I-R is not an exemption [52]. Further, it is one of the relevant oxidative pathways that could modulate I-R damage due to its close association with some oxidative components in this pathology [53]. Lipid ROS accumulation, which leads to oxidation and antioxidation activity mechanism via toxic lipid peroxidation, is a principal ferroptosis pathway that could be correlated with I-R [54, 55]. As an initiation mechanism, glutathione peroxidase (GPx) has an important antioxidant role in ferroptosis during reperfusion. Oxygenated blood promotes the stimulation of enzyme activity, primarily its isoform 4 (GPX4), which has the capabilities of a cytosolic antioxidant enzyme. This phenomenon then modulates the substrates of the lipoperoxide pathways such as H_2_O_2_, small hydroperoxides, and phospholipids that are inserted in the biomembranes [56, 57]. By contrast, arachidonic acid contains phosphatidylethanolamine, which plays a key role in the ferroptosis cell death signaling. Hence, it is a crucial target for modulating this oxidizing process [57–59].

## 5. Antioxidant Enzymes in I-R

Researchers are examining the safest and most effective therapies for regulating I-R damage. However, this tissue pathology is characterized by excessive oxidative damage that is challenging to resolve because free radicals can drive cells via different routes of cell death and subsequent necrosis [20]. Nevertheless, in recent years, a previous study about ROS has shown that these molecules are involved in different pathological processes closely correlated with I-R [60]. Therefore, antioxidant activity is the main therapeutic target of most pharmacological therapies to address this phenomenon [60]. However, not all therapeutic approaches have shown conclusive or favorable results. Thus, it is constantly necessary to make updates on antioxidant therapy in this event to improve the resolution of this pathophysiological condition [61]. The scientific community accepts the role of ROS and other important free radicals, such as superoxide radical (O2), formed by adding extra electrons in an oxygen molecule (OH), which is created from O_2_ via the interaction of H_2_O catalyzed by transition metals including iron in I-R [62]. Oxygen radicals can be formed by the action of singlet oxygen, which commonly occurs in ischemic tissues [63]. The eukaryotic cell has a defense system similar to that of enzymes, such as superoxide dismutase (SOD), catalase, glutathione peroxidase (GPx), and glutathione reductase, which inhibit reactive species formation [64, 65] For example, the concentrations of SOD1, SOD2, CAT, and GSH-Px decrease in I-R [66, 67]. Another important antioxidant complication is the reduction of GSH in the ischemic myocardium by buthionine sulfoximine, a cellular inhibitor of GSH, thereby making the tissue more susceptible to reperfusion damage [68]. One of the main concerns when choosing antioxidant enzymes as possible therapeutic targets is that the enzyme activity and the average concentration in tissue and the relationship they have in ischemia-reperfusion damage have not yet been correctly described or have not been conclusive [69].

## 6. Therapeutic Targets in Oxidative Damage

A detailed review of the therapeutic targets that can be a receptor for some antioxidant drugs should be performed. This allows redirecting research to the establishment of therapeutic alternatives that can have more interesting effects [70]. Calcium overload, which triggers the formation of reactive species in the mitochondria, and MPTP opening, which is involved in the release of Ca2^+^ from the mitochondrial matrix to the cytosol, are relevant [71]. Regulating the ferroptosis process, one of the key pathways in nonapoptotic death that is strictly correlated with cellular antioxidant capacity could be an interesting alternative for different pathologies involving I-R [64]. Undoubtedly, the modulation of ROS/RNS in the cytosol, which can prevent all types of cell damage, is a strategy that remains undiscovered due to a large number of possibilities [72]. Notably, future research must focus on the different types of tissues, variations in I-R injury, and modulating strategies.

### 6.1. Mitochondrial Receptors

Alternatives for modulating I-R damage should be identified. Oxygen depletion after ischemia is correlated with the inhibition of mitochondrial respiratory chain electron transport and the consecutive decrease in ATP levels, which leads to failure in Na^+^/K^+^ pump and Ca^2+^ accumulation [73, 74]. The electron transport chain can be the primary target of the mitochondria. This is confirmed by five main types of enzyme complexes, which are as follows: NADH-CoQ reductase, succinate-COQ reductase, CO-Q-cytochrome c reductase, cytochrome c oxidase, and ATP synthase, which are known in that order as complexes I–V and are all integrated into the inner mitochondrial membrane [75, 76]. Concurrently, ubiquinone and cytochrome c are the two freely diffusible molecules implicated in electron transfer between complexes previously mentioned [77]. Various signaling pathways, which are essential to normal cell function, require ROS activity from hydrogen peroxide, hydroxyl radicals, and superoxide anions. The main keys for triggering the formation of ROS are found in complexes I and III [78]. NADH commonly binds to the complex and promotes electron transfer flavin mononucleotide (FMN). Reduced levels of flavin decrease O_2_ superoxide concentrations and promote proton transfer that conducts ATP synthesis [78]. Mitochondrial complex II is the only enzyme that is part of both the Krebs cycle and electron transport chain. Succinate, which is oxidized to fumarate via the action of adenine flavin, mediated by the dinucleotide cofactor (FAD), is involved in this reaction [79]. Undoubtedly, mitochondrial complex II is a central modulator in metabolic and respiratory adaptation in response to different stimuli and abnormalities. Thus, it is a key receptor in the modulation of oxidative damage in I-R [80]. Previous reports have shown an overlap in respiratory complex II and mKATP channel agonists that can activate it. The association between mKATP and respiratory chain complexes has shown a correlation between complex II and decreased ROS production [81].

**Figure 1:**
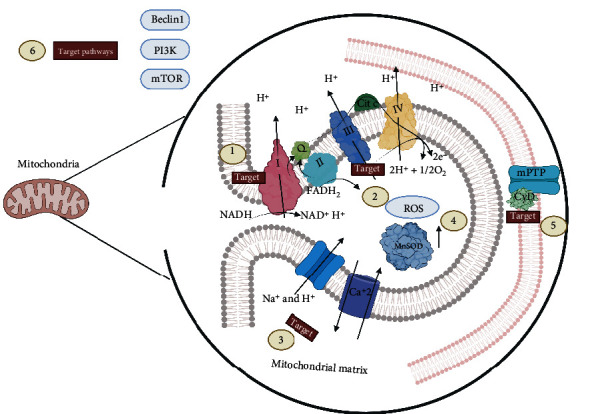
Mitochondrial targets for modulating oxidative damage induced by ischemia-reperfusion. The electron transport chain mainly in complexes I (1) and III (2) is the target of interest for reducing the formation of reactive oxygen species in the mitochondrial matrix as well as the activation of Na^+^/ H^+^ antiporters and calcium saturation (3) in the matrix along with the increased expression of antioxidant enzymes such as MnSOD (4) that carry out free radicals. In the mitochondria membrane, the blocking of MPTP by cyclophilin (CyD) is also essential (5), thereby preventing ROS from leaving the mitochondria to the cytosol. Beclin 1, PI3K, and mTOR (6), which are alternatives of interest for blocking mitochondrial damage, are some pathways that have good outcomes for reducing such damage.

The complex III Q-cytochrome c reductase molecule, which is implicated in the addition of four protons to the intermembrane space, is a significant site for ROS productions. The free radical ubisemiquinone leads electrons to oxygen, and this reaction results in a superoxide ion formation process that is enhanced by complex III inhibition [82]. Cytochrome c oxidase, better known as complex IV, mediates O_2_ reduction from H_2_O molecules by transferring four protons from the matrix into the intermembrane space, thereby increasing the electrochemical gradient and then entering as part of the intermediaries to this reaction [83, 84]. ATP synthase complex V promotes oxidative phosphorylation and induces ATP synthesis resulting in ATP formation [85]. Therefore, failure in the activity of this complex leads to inefficiency and dysregulation of mitochondrial function [86]. Complexes I and III are the principal targets because small amounts of free radicals are correlated with oxidative damage induction, mainly during hypoxia or ischemia. The inhibitors or modulators of these two molecules could manage hypoxic or ischemic conditions [87]. Interestingly, modulating mPTP opening is another interesting point for preventing ROS damage or even necrosis. This complex is a crucial effector in the cell death pathway. In addition, the activation of the mPTP function is the first step in the mitochondrial intrinsic necrosis pathway, leading to mitochondrial permeability transition and loss of inner mitochondrial potential [88]. Several pathways lead to the opening of mPTP. As a protein complex, they must interfere in one of its subunits, cyclophilin D (CyD), an essential modulator of mPTP. This makes it a key target for preventing cell death due to necrosis [89, 90]. A recent study focuses on the identification of novel compounds that can inhibit mPTP opening without any modulation of the CyD [91]. Mitochondrial ATP-sensitive potassium channels (mKATP) are opened after ischemia as a resolution measure, thereby modifying the activation of mPTP and delaying apoptosis. In addition, nonmitochondrial KATP can provide protective effects by promoting blood flow and excessive production of substrates [92]. These complexes promote the blocking of mitochondrial respiration and membrane disruption during diseases. Further, they are considered the primary cause of cell death in myocardial infarction I-R [93–95]. The mitochondrial antioxidant manganese SOD (MnSOD) expression is one of the objectives for modulating its dismutase scavenging function in superoxide radical O_2_ affecting several cell compartments. These are correlated with the pathophysiology of I-R, with the endoplasmic reticulum being sensitive to ROS, thereby making it responsible for maintaining calcium homeostasis [96, 97]. By contrast, autophagy is a crucial modulation target, which is responsible for cell recycling [98]. Previous studies have shown that this event contributes to the processes of cellular damage, and the key molecules are Beclin 1, mTOR, and PI3K [99, 100]. The mitochondria are important in pathological processes. To date, there is sufficient evidence about the morphological differences between the mitochondria, and that they are structurally and physiologically distinguished even in the same tissue [101] (Figure 1).

## 7. Molecular Targets for Ischemia in Different Tissues

Although the I-R phenomenon has many similarities in different tissues, the lack of oxygen is the leading cause of cellular imbalance. On the other hand, it is necessary to highlight the differential characteristics between tissues, mainly the critical therapeutic targets that will elucidate pathophysiological mechanisms and the design or implementation of new therapeutics or interventions. In this work, we selected some groups of tissues most affected by this phenomenon and try to highlight the molecular targets.

### 7.1. Myocardium

**Figure 2:**
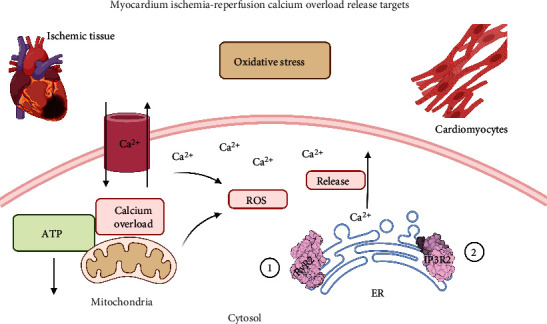
Calcium overload release targets. There are two principal ways of releasing Ca^2+^ accumulating in the mitochondria of the cardiomyocytes due to mitochondrial ROS formation. The first one is via type 2 ryanodine receptors RyR2 (1) and type 2 inositol 1,4,5-triphosphate (IP3R2) receptors (2). A mechanism that reduces ROS and Ca^2+^ cell damage.

The myocardium is an I-R susceptible tissue after epicardial coronary artery occlusion. The hypoperfused myocardial zone during myocardial infarction is a risk zone for oxidative damage and inflammation [102]. Clinical and preclinical research has shown a large number of cardioprotective agents, with mechanisms ranging from calcium overload to oxidative stress modulation. However, targeted therapy remains a challenge that has not been addressed altogether [103]. Calcium (Ca^2+^) released from the sarcoplasmic reticulum (SR) is important for excitation-contraction (E-C) coupling. The mitochondria, the major source of energy in the form of ATP, which is required for cardiac contractility, are closely interconnected with the SR, and Ca^2+^ is essential for the optimal function of these organelles. However, Ca^2+^ accumulation can impair mitochondrial function, leading to reduced ATP production and increased release of ROS. The calcium (Ca^2 +^) released by the SR is essential for cardiac excitation and contraction. ATP from the mitochondria is the main source of energy for the myocardial contraction process. However, the accumulation of mitochondrial Ca^2+^ affects the functioning of this organelle, which significantly decreases ATP and increases the formation of ROS [104]. Oxidative stress is directly associated with heart failure. Some studies have validated the role of Ca^2+^ in the development of this event, and it was found to be closely related to mitochondrial dysfunction [105]. Notably, there are two ways of releasing Ca^2+^ accumulating in the mitochondria of the cardiac cell, which are as follows: via type 2 ryanodine receptors RyR2 and type 2 inositol 1,4,5-triphosphate (IP3R2) receptors [106, 107] (Figure 2).

### 7.2. Hepatic I-R

**Figure 3:**
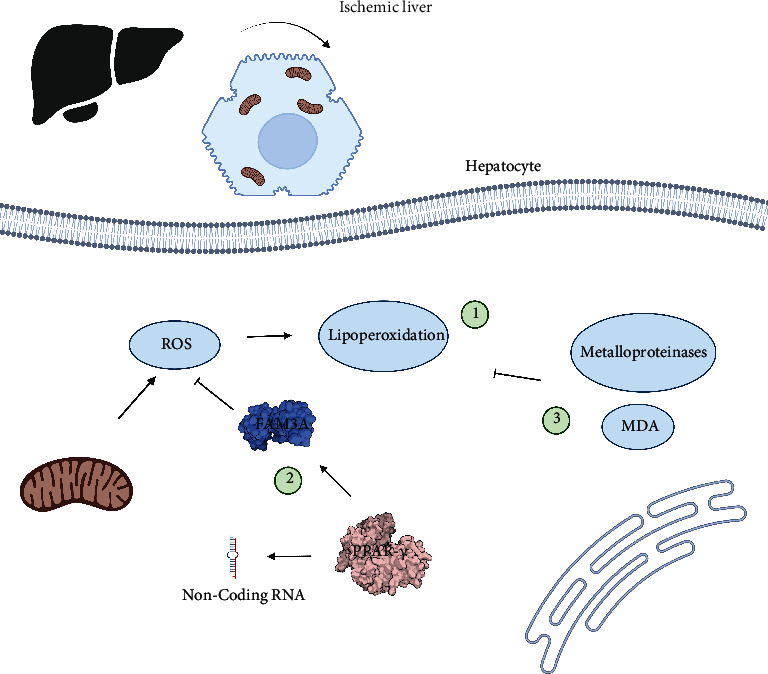
Main targets to regulate oxidative damage during hepatic ischemia. There are three principal points in hepatic tissue when oxidative damage is modulated. (1) Lipoperoxidation interferes with normal cellular functions and is the principal objective during hepatic I-R. That is why the peroxisome proliferator-activated receptor-gamma (PPAR-*γ*) is considered a good target. (2) It inhibits ROS production via the FAM3A complex and noncoding RNA. Besides, ROS formation of release blockade is crucial for diminishing lipoperoxidation, in addition, with the activity of metalloproteinases and malondialdehyde antioxidative complex.

The liver is extremely sensitive to oxidative damage caused by I-R. Therefore, blood flow must be restored to prevent or slow down cell death [108]. Some studies have reported the importance of ischemic preconditioning for the management of this pathology and the role of lipoperoxidation modulation in this mechanism. This explains why peroxidation signaling pathways are relevant in reducing this condition [109]. By contrast, it is important to identify the role of peroxisome proliferator-activated receptor-gamma (PPAR-*γ*). That is, it inhibits the production of ROS in a pre- and posttransductional method, via the FAM3A complex and noncoding RNA axis, as reported by several workgroups [110]. In addition, the other important targets for modulating oxidative damage are metalloproteinases and malondialdehyde, which are the enzyme complexes involved in the cellular oxidative process [111, 112] (Figure 3).

### 7.3. Renal Tissue

**Figure 4:**
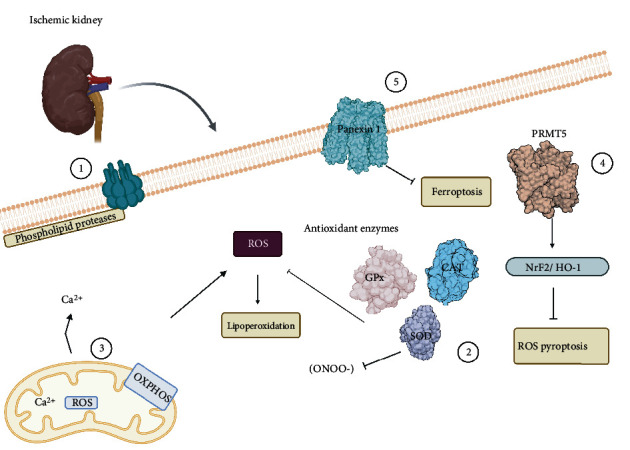
Relevant targets during ischemia-reperfusion in renal tissue. The main ways to decrease renal oxidative damage during I-R are (1) activation of phospholipid proteases, (2) the increment of antioxidant enzyme complex reducing free radicals and blocking lipoperoxidation, (3) modulation of Ca^2+^ and ROS mitochondrial release as well as controlling oxidative phosphorylation, (4) PRMT5 protein arginine methylation transferase is a regulation mechanism to stop ferroptosis, and (5) the membrane receptor Pannexin 1 through that blockaded ROS pyroptosis through Nrf2/HO-1 signaling pathway.

The kidney is a specific organ that can be affected by I-R, which could lead to irreversible kidney injury. However, renal occlusion is inevitable during transplantation. That is why there are countless models for this phenomenon [113]. These advancements are crucial in understanding the pathophysiology of renal I-R, and they propose some therapeutic targets that can improve management. It is important to provide an overview of the possible therapies and receptors that can reduce oxidative damage in the kidneys [113, 114]. Similar to other organs, ROS plays a fundamental role in oxidative stress, which changes mitochondrial oxidative phosphorylation, ATP depletion, an increase of intracellular calcium, and activation of membrane phospholipid proteases, processes that could have results as a therapeutic alternative [115]. The interesting molecules to modulate the damage induced by renal I-R are more aimed at increasing the expression of antioxidant enzymes or their activity to offer a resolution of oxidative damage [116, 117]. Therefore, some interesting targets are the SOD, CAT, and GPX receptors, since, without a doubt, the neutralization of ROS and hydroperoxides mediated by these enzymes is a probable target for the treatment of renal I-R [118]. Notably, although antioxidant therapy is a viable alternative, damage caused by I-R cannot be fully treated. However, its efficiency is sufficient to considerably reduce oxidative damage [62, 118]. Some studies have shown the beneficial effects of free radical scavenging molecules on renal I-R. Molecules including melatonin can modulate the damage induced by renal reperfusion in ischemic kidneys due to its antioxidant activity [119]. Moreover, lipoperoxidation is a proven mechanism with good outcomes in renal tissues, where we could highlight the increase in SOD activity as the main target [120]. Furthermore, Diao et al. showed that the inhibition of protein arginine methylation transferase 5 (PRMT5) blocked ROS-mediated pyroptosis via the Nrf2/HO-1 signaling pathway. Therefore, PRMT5 is an interesting management target in renal I-R injury [121, 122]. The mitochondrial receptor MnSOD, an antioxidant enzyme capable of scavenging O_2_ free radicals, while controlling peroxynitrite radical (ONOO-), can successfully modulate I-R in renal tissues [123]. Although the role of ferroptosis in the renal I-R phenomenon has not been completely elucidated, several molecules can be targeted and provided interesting possibilities for therapeutics [124]. Pannexin 1 is an ATP-releasing protein that exhibits proapoptotic properties in renal I-R [125]. With consideration of molecular targets for modulating ferroptosis, the GPX4 enzyme can be a key regulator of lipoperoxidation [126]. Therefore, its activation is strictly correlated with the process of cell death and, consequently, the accumulation of ROS. This mechanism was found to be successful in pharmacological alternatives including irisin [127] (Figure 4).

### 7.4. Brain Tissue

The brain is the most sensitive organ to blood supply interruption without the possibility of repair in I-R. That is, 20 minutes of ischemia is enough to exceed the threshold of damage it can withhold [18]. That can potentially cause or lead to oxidative stress-induced behavioral and cognitive decline. Oxidative stress in the brain caused by I-R leads to the primary etiologies of brain damage and significant neuronal effects, resulting in tissue destruction and cell death. These include lipid peroxidation, protein denaturation, inactivation of enzymes, nucleic acid, and DNA damage, the release of Ca^2+^ from intracellular stores, damage to the cytoskeletal structure, and chemotaxis [128]. Phospholipids in the brain are vulnerable to ROS-mediated peroxidation. However, proteins and DNA are targeted by ROS, and they become problematic with aging as aging brains exhibit high oxidative stress-induced mutation levels in the mitochondrial DNA [129, 130].

Perhaps, we cannot find an effective therapeutic target for reducing this damage. That is why ischemic preconditioning combined with antioxidant therapies will most likely be critical regulators for ischemic stroke [131]. Current studies have focused on the pathways of oxidative stress that involve a variety of cellular pathways, receptors, and processes that can be used on focused therapy for oxidative damage, such as autophagy, mitophagy, and necrosis, which are involved in eliminating excess ROS and subsequent cell death triggered by these free radicals [132, 133]. The endogenous protective mechanisms in the brain included the antioxidant enzyme systems and the low-molecular-weight antioxidants [134]. In response to stress, cells increase their antioxidant defenses with nuclear factor erythroid 2-related factor (Nrf2), an important transcription factor [135]. Therefore, Nrf2 has been proposed as a pharmacological target in pathologies with oxidative features since it modulates several genes encoding antioxidants and detoxification enzymes such as heme oxygenase 1 (HO-1), NAD(P)H dehydrogenase quinone 1, superoxide dismutase 1 (SOD1), glutathione peroxidase 1 (GPx1), and catalase (CAT) [136]. By contrast, mitochondrial dysfunction suggests several diseases, including neurodegeneration [137].

The mitochondrial role in ischemic shock and its pathogenesis mainly involves the formation of free radicals [138]. mtDNA is particularly susceptible to oxidative damage because of its proximity to high levels of mitochondrial ROS production and its relatively poor defense against damage. Healthy mitochondria contribute to oxidative stress resistance by increasing respiratory capacity [139]

**Figure 5:**
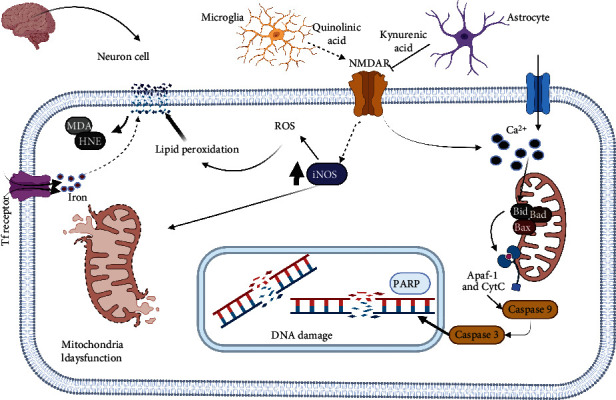
Oxidative stress in neuron cells. Several mechanisms activate oxidative stress during ischemia in neuron cells, principally leading by the quinolinic acid released by microglia and mitochondrial Ca^2+^ overload and ROS formation after BLD, BAD, BAX complex, and APAF-1 and CytC activate caspases pathways that result in DNA damage. Nevertheless, astrocytes intend to block that oxidative stress and lipoperoxidation that could damage plasmatic membrane releases kynurenic acid. Unfortunately, Tf receptors promote cell damage through ferroptosis. Therefore, all of these would function as excellent regulatory points for I-R oxidative injury in nervous tissue.

Taken together, ATP synthase and the electron transport chain make up the OxPhos system, which is the leading promoter of the mitochondrial electrochemical gradient [140, 141]. Crucial key points for electrons to enter are complex I and II of the electron transport chain, which through ubiquinone transfer electrons to complex III and this in turn to complex IV. Proton pumping via the mitochondrial membrane is the primary mechanism for maintaining the membrane potential. These protons are then used by complex V ATP synthase to form ATP and complete the oxidative phosphorylation process [142]. Reversible phosphorylation mechanisms are relevant targets of this whole process. Preserving these phosphorylation epitopes could offer a regulatory control for reducing oxidative damage since it could allow the regulation of OxPhos mediated by calcium and the ADP-shuttle mechanism [143]. The OxPhos complexes are phosphorylated in vivo by the second messenger Ca^2+^, thereby triggering the phosphorylation of most mitochondrial proteins, a process mediated by calcium-dependent phosphatases during ischemic stress [144]. This phosphorylation alters the electron transfer kinetics, which affects the allosteric regulation of ATP and ADP [145]. The mitochondrial membrane potential (ΔΨ*m*) becomes positive in the inner chamber during oxidation, and cytochrome c (CytC) is released into the cytoplasm. The release of CytC from the mitochondria is an important pathway for the cascades of apoptotic events [146]. These proapoptotic proteins, such as Bid, Bad, Bax, Bak, Bok, and Bim, in the outer mitochondrial membrane, increase the permeability of membranes, thereby forming specific pores and stimulating free CytC release. Third, CytC binds to apoptosis protein-associated factor 1 (Apaf-1) and forms the Apaf-1/caspase-9/CytC complex. Finally, caspase-3 is activated, which triggers apoptosis and delays neuronal death [147]. Lipid peroxidation is one of the significant consequences of ROS-mediated injury to the brain. This ultimately leads to the production of conjugated diene hydroperoxides that attack lipids containing carbon-carbon double bond(s) in specific polyunsaturated fatty acids (PUFAs). Among these compounds, malondialdehyde (MDA) and HNE are the breakdown products of lipid peroxidation, and they are elevated in patients with ischemic stroke [148], with infarct size, stroke severity, and patient outcome. MDA can be the most mutagenic lipid peroxidation product, and HNE is the most toxic [149]. MDA is widely used as a biomarker for lipid peroxidation of omega-fatty acids, HNE is a cytotoxic product originating from peroxidation, and it is considered as one of the significant toxic products generated from lipid peroxides. The highly toxic characteristic of HNE can be explained by its rapid reactions with thiols and amino groups [150]. HNE is a bioactive marker of lipid peroxidation and is a signaling molecule involved in the regulation of several transcription factors, such as nuclear factor erythroid 2-related factor 2 (Nrf2), activating protein-1 (AP-1), NF-*κ*B, and peroxisome proliferator-activated receptors (PPAR), cell proliferation and differentiation, cell survival, autophagy, senescence, apoptosis, and necrosis [151]. Hemoglobin (Hb)/haem is a putative neurotoxin. Hb is the most abundant protein in the blood and is released from lysed red blood cells after stroke. It can be engulfed by the microglia in the perihematomal zone and metabolized into ferrous/ferric iron, which induces ROS formation and lipid peroxidation [152]. The excess ferrous iron accumulates in the neurons via the transferrin (Tf)–Tf receptor system that forms highly toxic hydroxyl radicals (^∙^OH). These hydroxyl radicals attack DNA, proteins, and lipid membranes, leading to the disruption of cellular function. Ferroptosis was found in organotypic hippocampal slice cultures exposed to glutamate [153]. It can be distinguished from other types of regulated cell death because it does not require caspases ATP depletion or mitochondrial ROS generation (Bax/Bak) or elevations in intracellular Ca^2+^ levels [154]. Ferroptosis is triggered by glutathione biosynthesis or glutathione peroxidase 4 (GPX4) activity inhibition and is associated with shrunken and electron-dense mitochondria morphologically [155]. Tryptophan (TRP) is an aromatic essential amino acid whose route of TRP metabolism is the kynurenine (KYN) pathway, and the primary end products are nicotinic acid and its derivatives and NAD^+^ and NADP, which are two ubiquitous coenzymes In this catabolic process, starting from the central compound, kynurenine (KYN) forms kynurenic acid (KYNA), xanthurenic acid (XA), and picolinic acid [156] (Figure 5). KYNA is produced mainly in astrocytes, and quinolinic acid (QUIN) degradation occurs in microglial cells in the central nervous system. More recently, tryptophan oxidation via the kynurenine pathway has been implicated in inflammation and oxidative stress in the brain that occurs after stroke [157]. Elevated QUIN levels can cause excitotoxic cell death. The hippocampus and striatum are most sensitive to QUIN neurotoxicity. QUIN can directly interact with free iron ions to form toxic complexes that exacerbate ROS formation, oxidative stress, and excitotoxicity [158]. Moreover, it induces lipid peroxidation, produces ROS increases iNOS expression, decreases SOD activity, and causes mitochondrial dysfunction QUIN which stimulates mitochondrial dysfunction and apoptosis [159]. By contrast, the advantage of KYNA is that it cannot be metabolized to excitotoxic agents and scavenges oxygen radicals, thereby decreasing cellular damage. The application of KYNA in high concentrations or for a prolonged time causes neuronal cell damage [160]. The multiple effects of the kynurenine pathway and its changes during stroke have increased in recent years, thereby allowing interference with therapeutic targets. DNA damage includes oxidative modification and endonuclease-mediated DNA fragmentation. DNA oxidation may activate repair enzymes, such as poly (ADP-ribose) polymerase (PARP). PARP activation progresses from neuronal elements and localization of infiltrating inflammatory cells 3–4 days after stroke. The activation of PARP leads to DNA injury in the brain [161]. Indeed, there is a strong association between oxidative stress and PARP activation in the brain, and oxidative stress in the neurons can induce PARP activation [162]. PAR can directly affect mitochondrial membrane potential collapse [163]. Thus, PARP-1 activation may inhibit glycolysis and cause energy depletion, thereby leading to altered cellular metabolism.

### 7.5. Lung Tissue

**Figure 6:**
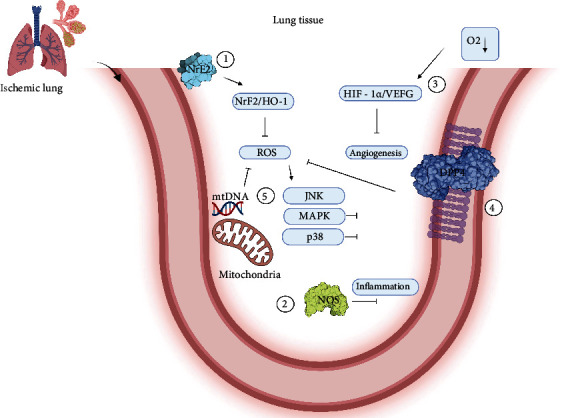
Molecular targets to decrease oxidative damage during I-R. (1) One of the main points to reduce oxidative damage is the NrF2 factor, the starting point of the NrF2/HO-1 signaling pathway that triggers ROS blockade. (2) Nitric oxide synthase, being blocked, can prevent the activation of the inflammatory cascade. (3) Low oxygen concentrations in the cell lead to the activation of HIF1 and HIF 1*α*/VEGF, one of the primary mechanisms for generating new vasculature. (4) DPP4s decrease oxidative stress when their expression increases. (5) In addition, mitochondrial DNA can function as a signaling mechanism that allows the activation of programs to block the production of reactive oxygen species.

The lungs are affected by I-R indirectly. Several signaling pathways such as Nrf2/HO-1 and HIF 1*α*/VEGF have protective effects on this organ [164]. NrF2 is a transcriptional factor that protects cells from stress, and oxidative processes activate NrF2 to initiate such an effect. In turn, HO-1 becomes a rate-limiting enzyme that can reduce oxidative stress by increasing its expression in the lung either via local or peripheral ischemia [165, 166]. Hypoxia-inducible factor 1-*α* (HIF-1*α*) and cell repair mechanisms mediated by VEGF are implicated in the regulation of angiogenesis in ischemic events [167, 168]. After ischemia damage to the lungs, there is a significant loss of plasma proteins and inflammatory cells, and there are high amounts of HIF-*α* and its regulatory target VEGF during I-R in local tissues [169]. Moreover, recently, the close association between these two molecules has been correlated with repair mechanisms independent of angiogenic activity [164]. The mitochondrial approach may also be a good alternative to modulating oxidative damage. Some reports have shown interesting results regarding protecting the integrity of the mitochondrial DNA using the oxidative approach. That is, mtDNA could serve as a sentinel of ROS-mediated functions, as observed primarily in the lung tissues [170]. Using conventional oxidative stress as a therapeutic target in ischemia could be complicated. Therefore, preconditioning alternatives can be another therapeutic option. Researchers have designed in vitro and ex vivo experimental data in which the tissues and cells are exposed to high concentrations of polyethylene glycol-catalase (PEG-CAT) to protect against cytotoxicity caused by oxidative stress. This mechanism then preserves cellular metabolism and mitigates pulmonary I-R. Therefore, PEG-CAT can be an important therapeutic target [171]. Anti-inflammatory approaches for decreasing pulmonary ischemia remain unclear. It was proposed that the establishment of novel therapeutic strategies should involve the inhibition of transcriptional factors that activate oxidative stress with better techniques. For example, MAPKs that are activated after oxidative stress in the inflammatory models of pulmonary ischemia and different signaling pathways, such as p38, c-jun N-terminal kinase, p38 inhibition, or JNK, have protective effects in this organ [172]. In addition, ROS and RNS mediate inflammatory reactions by activating alveolar macrophages. With the activation of the inflammatory cascade, multiple potential ROS generators such as the mitochondria, xanthine oxidase, NOX, NOS uncoupling, and neutrophils must be considered as a therapeutic target for oxidative damage [173]. By contrast, the expression of DPP4 is directly correlated with decreased oxidative damage. That is, the capillaries are the main concentration regions of this expression, and DPP4, a serine protease, commonly cleaves the substrates with proline and alanine in the latter position [174] (Figure 6).

### 7.6. Skeletal Muscle

**Figure 7:**
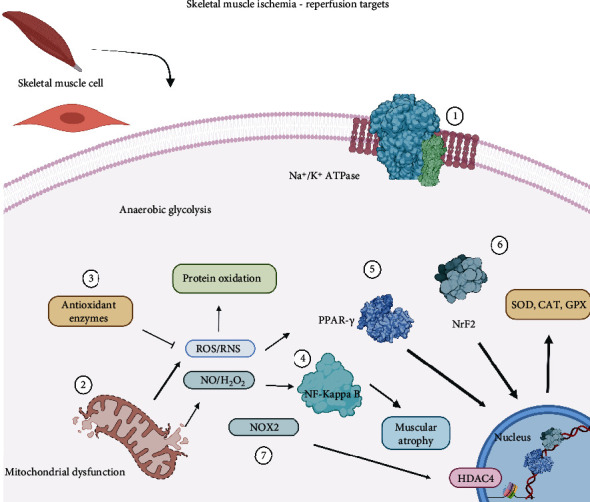
Skeletal muscle I-R targets. (1) Na+/K+ ATPase activation promotes the saturation on Na+ and K+. That is why modulation of these enzymes could be an excellent alternative to reduce damage. (2) Blocking mitochondrial release of ROS and RNS during dysfunction could improve cellular damage. (3) Scavenger activity of antioxidant enzymes is one of the main alternatives to modulate this condition. (4) Activation NF-*κ*B leads to muscular atrophy for several inflammatory mechanisms. (5) PPAR-*γ* translocations, as well as NrF2 (6), result in the expression of the antioxidant complex. (7) NOX2-depending pathways conduce to activation of HDAC4 that facilitates gene expression.

In the limbs, skeletal muscle is the predominant tissue, and pathophysiological literature indicates that the damage threshold of this tissue is exceeded after 3 h of ischemia and is irreversible at 6 h [175]. Some studies about I-R showed that the main mechanisms of cell damage and death are mitochondrial dysfunction and mitochondrial proapoptotic protein release [176]. Similar to other cells, I-R in the myocyte is mediated by the mitochondrial membrane potential and the proton gradient that promotes ATP synthesis via oxidative phosphorylation [177]. This reduction during the ischemic process promotes ATP synthesis and inhibits Na^+^/K^+^ ATPase, thereby increasing intracellular Na^+^ and Ca^2+^ and anaerobic glycolysis. Further, the mitochondria play an important role in the pathophysiology of I-R in this tissue, and the free radicals generated by the skeletal muscle during rest and activity are NO and superoxide, which is dismuted into H_2_O_2_. However, there are still several limitations, and few studies have identified the nature of ROS or RNS present in the muscle fibers. Most reports have only examined cell surface free radicals [152]. Consequently, there are only a few reports about NO or H_2_O_2_ or substances that can cross mitochondrial barriers, but there are a large number of reactive species that have not been confirmed to be involved in skeletal muscle physiopathology [152]. To avoid deleterious effects on tissues, there are several cellular mechanisms to modulate free radicals such as the mitochondrial and cytosolic isoforms of superoxide dismutase (MnSOD and CuZnSOD) in addition to CAT and GPX modulation of their expression [178]. Any cellular processes are regulated by ROS and RNS, such as the activity of transcriptional factors, ionic transportation, apoptosis, and metabolism [179]. Proteins in skeletal muscle are susceptible to oxidation of their sulfhydryl groups or the formation of disulfide bonds. These processes are involved in the modulation of protein functions [180]. In the same way, ROS also functions as excellent second messengers in the activation of apoptosis programs such as the NF-kappaB pathway, which is involved in muscle degeneration and atrophy [181]. Several alternatives of regulation, such as the PAR-gamma coactivator-1 alpha (PGC1-*α*) pathway, are redox-sensitive, in which ROS would play a regulatory role [182]. In addition, ROS dependent on Nox2 is involved in the regulation of histones such as histone deacetylase 4 (HDAC4), this happens during vigorous muscle activity, as a regulation mechanism [183]. Not less important is the already described NRF2 transcriptional factor involved in multiple regulations of antioxidant defense [184]. Under conditions of oxidative stress, NRF2 is found in the cytoplasm thanks to the activity of degrading proteins; after it is released, it translocates into the nucleus where it activates the transcription of antioxidant gene programs and their respective protein [185]. Although it well tolerates oxidative damage, these modulation strategies are essential for the resolution of countless pathologies correlated with ischemia [186] (Figure 7)

## 8. Ischemia-Reperfusion Antioxidant Pharmacodynamics

Antioxidant therapy has been used to modulate oxidative stress in different experimental models. Generally, some proven strategies are used as antioxidant preconditioning without completely effective outcomes [7, 187, 188]. This can be explained by the nonselective characteristic of ROS modulation, which directly interferes with cell signaling pathways [189]. This has led to alternative approaches such as activation of the Nrf2 pathway by fumaric acid derivatives, resulting in a proven antioxidant activity [190]. Another relevant strategy is using ROS-producing enzymes such as Nox and MPO, which induce a more specific response by modulating pathological conditions [191, 192]. However, the most promising approach involves enzyme activity, particularly via drugs with a potential to reverse eNOS activity in pathologies correlated with oxidative stress [193]. However, despite advancements, almost all innovative cardiovascular therapies have been inadequate in the management of these pathologies.

### 8.1. Free Radical Scavengers

Notably, reactive species at low concentrations fulfill cellular functions as metabolic bioproducts or second messengers. At high concentration, they have deleterious effects, mainly in pathological events such I-R [194]. These effects conceptualize as oxidative stress and lead to the opening of mPTP, resulting in protein and DNA damage [195]. ROS signaling can be interfered with via the inhibition of complex I, using drugs such as metformin. This then reduces the amount of ROS in the cytosol [196].

### 8.2. Mitochondrial Respiration Chain Blockers

**Figure 8:**
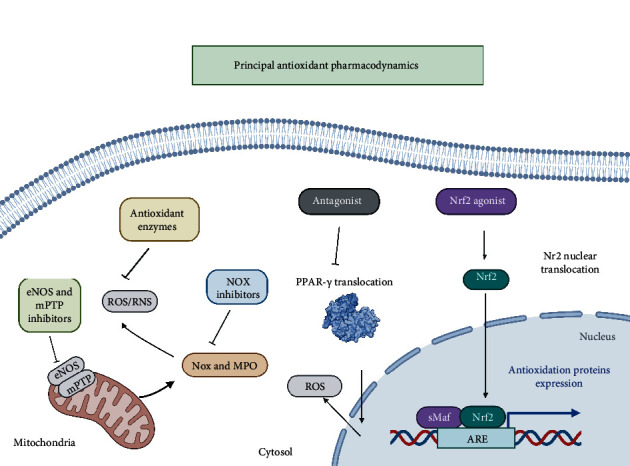
Principal antioxidant pharmacodynamics. The enlisted mechanisms are promising targets that are part of the pharmacodynamics of several drugs used in clinical practice. Antioxidant enzymes as a scavenger to reduce ROS and RNS, NOX inhibitors blocking free radical production, eNOS and MTTP inhibitors leading to reduction of reactive species and avoiding is release. Nuclear factors agonism and antagonism regulate the antioxidant expression and ROS production.

The electron transport chain stands out during the reperfusion process. This explains why direct modulation can be an alternative for reducing ROS production and the consequent activation of mPTP with cell death as an outcome [197]. The deregulated production of ROS in the mitochondrial respiratory complexes is associated with the I-R process. Regulating these processes modifies the harmful nature of ROS to a protective one, and these respiratory complexes are the main targets to carry this out [198]. Complexes I and III are major superoxide production sites. Electrons are transferred along the chain and back to complex I where NAD+/NADH is reversed and ROS production increases [199]. Using this approach, several drugs have been tested under I-R conditions, thereby providing varying but important results for understanding the phenomenon [200–203]. Some reports used the reversible inhibition of transiently inactivated complex I to diminish the generation of ROS without losing its function [59, 204, 205]. In these categories, some compounds such as biguanides, amobarbital, nicorandil, rotenone, and S-nitroso-2-mercaptopropionyl glycine have shown interesting results [205–210]. Highlighting metformin has exhibited cardioprotective properties by modulating complex I at high doses [211, 212]. Some strategies use acidic citric intermediates, malate, and oxaloacetate to inhibit complex II. Although it is not a specific site for the formation of ROS during the reperfusion process, it is correlated with complex I and III modulations, which allows cardioprotection regardless of K^+^ concentrations [213, 214]. Notably, some important mechanisms are not directly involved in the production of ROS nor as second messengers in the adaptation mechanism to hypoxia [215]. These signaling pathways are directly linked to ischemic preconditioning in different pathological conditions [216]. A consecutive modulation of complex III via the ubiquinol oxidation center (Qo site) has shown cardioprotection [217]. Reduced cytochrome c activity has a similar effect in electron transport from complex III to IV, an event that reduces superoxide-free radical production, which is a poorly understood mechanism [218] (Figure 8).

### 8.3. MPTP Inhibitors

The importance of MPTP for the development of I-R-mediated oxidative damage has been discussed. They represent a key point for the release of ROS/RNS from the mitochondria [36]. However, the therapeutic target approach of this protein complex is via its subunits, with CyD as one of the main ones, to which countless drug prototypes have been designed [219]. Nevertheless, in vivo and clinical data are not favorable enough to establish a therapy [220, 221]. Some analog drugs of CyA have shown favorable outcomes in myocardial, hepatic, and cerebral animal models [222]. Even some drugs that are not CyA analogs have interesting outcomes via this pathway in vivo models [223]. Other than this therapeutic target, other possible alternative therapies such as N-phenylbenzamides and cinnamic anilides can inhibit mPTP activity, thereby providing protective effects against oxidative damage [224, 225]. Nrf2 and NF-*κ*B regulators are also good alternatives to ameliorate oxidative damage via this pathway [226–228] (Figure 1).

### 8.4. PPR's Gamma Inhibitors

Peroxisome proliferator-activated receptor gamma (PPAR-*γ*) is the target of multiple studies about cardiovascular pathologies [229]. Several isoforms have been described. However, the *γ* isotype is the most relevant in these I-R-related diseases [230]. After binding to endogenous ligands, the retinal X receptor is heterodimerized with a nuclear receptor, thereby inducing or repressing gene expression [231]. Therefore, PPAR has relevant roles in hepatic IR injury [232]. Some angiotensin II drugs are associated with this receptor, which exhibits an inhibitory effect [233, 234]. Several drugs attenuate PPAR I-R via antagonism, thereby reducing ROS production.

### 8.5. RNAS Transcripts in I-R

**Figure 9:**
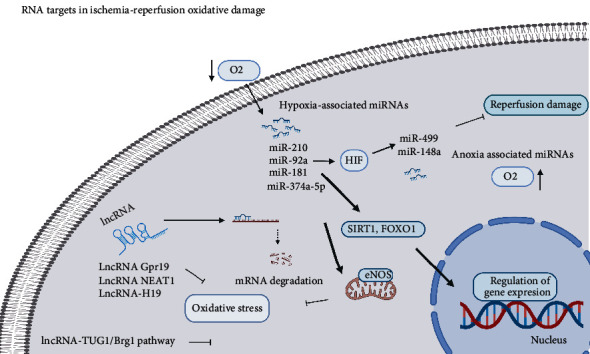
RNA targets in ischemia-reperfusion oxidative damage. We can make a group of the different miRNAs among those directly associated with the decrease in oxygen or hypoxia that directly activate the expression of HIF or are sensitive to it. Noncoding RNA activates various transcription factors to regulate the gene expression of antioxidant enzymes and prevent the formation of reactive species that exacerbate oxidative stress. Furthermore, those related to reperfusion or anoxia mechanisms are responsible for the resolution or inflammatory mechanism. Similarly, various lncRNAs block signaling pathways to reduce oxidative stress.

RNA and DNA are targets for modulating gene expression. Currently, advancements in molecular biology can allow them to be used as excellent therapeutic targets in multiple pathologies [235]. For example, the modulation of antioxidant enzymes using gene therapy has been useful in oxidative stress if a specific target receptor is already known [236]. Redox homeostasis has a direct correlation with cell function. Redox imbalance leads to oxidative stress production, which inhibits the development of vascular diseases and I-R injury, as well as triggers transcriptional and posttranscriptional modulation in gene expression [237, 238]. In addition, hypoxia is considered an important stimulus to regulate microRNA (miRNAs) expression [239]. Some miRNAs, known as hypoxia MIR, are even associated with hypoxia, and some of these transcripts are involved in the pathophysiology of ischemic and cardiovascular diseases [240]. An example is miR-210, a hypoxia-inducible transcript that promotes cell survival and improves cardiac function via antiapoptotic and angiogenic mechanisms [241]. Moreover, HIF is regulated by these transcripts with an important role in these pathologies [242]. Other examples of these nuclei of response to oxidative damage are NRF2, FOXO1, and NF-kB in the inflammatory part [243–247]. And the widely studied transcriptional factor p53, among the multiple stimuli that activate it, is ROS one of them, which consecutively triggers proapoptotic and antiproliferative mechanisms [248]. Even a single oxidative stress stimulus, such as H_2_O_2_ and O_2_, could be correlated with complex redox imbalance mechanisms in pathologies such as aging and limb ischemia [249, 250]. However, even though the role in oxidative stress of noncoding transcripts (miRNA and lncRNA) is widely known, their use as a therapeutic strategy remains premature [251, 252]. miRNAs are RNAs that regulate gene expression by forming hybrids with mRNAs, altering their translation [253]. Moreover, they have a regulatory role in oxidative stress via their interactions with SIRT1, FOXO1, and eNOS [254]. Furthermore, in different pathologies correlated with oxidative stress, these regulatory mechanisms have begun to take center stage [224] In I-R, miRNAs have great advantages when used as a therapeutic target. In the future, they could be considered pharmacological approaches in clinical practice [255]. Noncoding RNA similar to miR-92a has a proangiogenic effect, and miR-499 can decrease damage induced by hypoxia-reoxygenation. miR-24 had a similar mechanism correlated to the attenuation of infarct size in animal models [256–258]. Recently, the use of novel noncoding RNAs as therapeutic alternatives is emerging. For example, miR-181 is a drug target with an effect on experimental myocardial I-R [259]. miR-148a alleviates hepatic I-R and is implicated in resolution pathways [260, 261]. Another alternative is miR-374a-5p. This decreased myocardial cell damage in an I-R model [262]. Small RNAs are not the only noncoding transcripts relevant to disease-related I-R. Long RNAs (lncRNAs) are expressed by an opposite strand of mRNA, and they are located in the introns of annotated genes and transcribed from enhancer regulatory elements (eRNA) [263]. Moreover, this transcript mechanism varies, ranging from repressors to activators of gene expression, or even posttranductional regulators. They are also mRNA splicing and stability modulators; lncRNAs play an important role in regulating the response to oxidative stress [264]. For example, lncRNA Gpr19 inhibits and attenuates (I-R) injury after acute myocardial infarction, and lncRNA NEAT1 alleviates sepsis-induced myocardial injury by modulating oxidative stress [265, 266]. Some drugs including propofol have shown a beneficial effect against I-R oxidative stress under different conditions via the lncRNA-TUG1/Brg1 pathway in liver cells in an IR model [267]. Drugs such as metformin regulate oxidative stress via transcripts such as lncRNA-H19 [268]. In a recent study, ZFAS 1 lncRNA was found to reduce ischemic stroke via the regulation of some oxidative stress mechanisms [269]. (Figure 9).

## 9. Alternative Interventions in Clinical Trials

Regardless of the several researches carried out to decrease damage caused by I-R injury, there is still no relevant pharmacological therapy that reduces damage caused by this condition in clinical practice. However, some studies have shown important data, particularly about those regarding Cyst A, in advanced pharmacological phases or pilot studies [270]. By contrast, metoprolol, a *β*-blocker, exhibited interesting activity in patients with I-R induced by myocardial infarction [271, 272]. Moreover, some glucose modulators have inhibited I-R damage via several mechanisms, with glucose/insulin/potassium (GIK) as the primary on [273]. In terms of clinical results, patients with acute coronary syndrome have presented with a significant reduction in infarct size [274]. In addition, intracoronary administration combined with thrombectomy significantly reduced infarct size [275]. The application of novel therapies in clinical practice is challenging, and there is an extensive list of not favorable outcomes in I-R treatments in clinical trials. One of the main reasons for these failures is the lack of clarity and significance in preclinical trials, thereby making it impossible to obtain relevant clinical information [276]. The most relevant examples are studies about inhaled nitric oxide and sodium nitrite for myocardial infarction. These strategies were tested in clinical practice. However, they were not effective in reducing infarct size [277]. The use of TRO40303, an MPTP inhibitor, had unfavorable outcomes in the MITOCARE study [253]. In the TREAT study, the outcomes of ticagrelor and clopidogrel treatment in similar pathological conditions were not significant [278]. However, some reports highlight interesting epitopes or therapeutic approaches, thereby allowing the appropriate incorporation of new clinical evidence into the practice guidelines of phase III trials, which are required to assure a solid preclinical background (Table 1).

## 10. Perspective

The therapeutic targets most likely to be extrapolated in clinics settings are not yet fully elucidated. Hence, further studies should be performed to assess relevant epitopes. Most approaches used to reduce I-R damage are inflammatory. Therefore, many pharmacological effects of the peroxidative type have not been considered, thereby establishing the grouping of the said markers and targets of oxidative damage. After elucidating the molecular targets implicated in their physiopathology, a realistic approach in all pathology therapies is a pragmatic approach in all pathology therapies. The direct modulation of calcium overload in the cytosol can be a common strategy and, undoubtedly, a key element in stabilizing cellular pH. By contrast, the uptake of ROS mainly via scavenger molecules that may reduce damage can also be an exciting alternative.

## 11. Conclusion

I-R injury caused by the interruption of oxygen flow and the consecutive restoration of oxygen concentration, which is known as reperfusion, is still poorly understood [279]. These therapeutic targets can be a great alternative for modulating I-R injury. Reducing oxidative damage in cells could address several pathologies associated with I-R. Redox signaling is one of the processes with several therapeutic targets. However, most interventions present extremely ambiguous pharmacodynamics [280]. Thus, it is essential to elucidate the specific molecular mechanisms by which pharmacological interventions work. The mitochondria play a key role in the development of these cellular signaling [281]. Therefore, researchers are interested in approaches with correlated epitopes. Although the preclinical outcomes of targeted therapies are favorable, they have not yet been applied in clinical practice, and their adverse effects were not evaluated. Hence, pharmacological repositioning is a good alternative at present [282]. However, it is necessary to develop new active principles with a specific activity to resolve this pathology or to consider therapeutic combinations via in vitro and even in vivo tests [283]. Nevertheless, new active compounds with specific activity must dress to resolve pathologies or take advantage of the drug's antioxidant properties that can be considered adjuvant therapies in clinical settings. Proposing new therapy is necessary. This phenomenon is related to multiple pathologies and surgical procedures in oxidative stress and alterative to modulate ischemia-reperfusion.

## Figures and Tables

**Table 1:** Pharmacological approaches against I-R pathologies.

Family	Pharmacodynamic	Results
*β*-Blockers	Reduce the cardiac frequency and calcium overload blockade	Regulate myocardial infarction
Glucose modulators	Regulate glucose/insulin/potassium concentration	Reduce myocardial infraction and infarct size
Immunomodulators (abciximab)	Reduce Inflammation and oxidative stress activation	Reduce infarct size in acute coronary syndrome
Inhaled NO and NaNO_2_	Regulates oxidative stress	Failure in reducing myocardial infarction
MPTP inhibitors	Blockade of mitochondrial ROS release	Adverse effects and not significant data
Statins	Oxidative scavengers and IL10 expression	No significant data in acute coronary infarction
ARA II	PPAR-*γ* expression and antioxidant activity, SOD2 expression	Significant data were preventing I-R

## Data Availability

We provide editable versions of our figures and editing platform publishing license.
